# Thermographic Behavior of the Cornea During Treatment With Two Excimer Laser Platforms

**DOI:** 10.1167/tvst.10.9.27

**Published:** 2021-08-24

**Authors:** Alberto Haber-Olguin, Eduardo J. Polania-Baron, Francisco Trujillo-Trujillo, Enrique O. Graue Hernandez

**Affiliations:** 1 Department of Cornea, External Disease, and Refractive Surgery, Instituto de Oftalmología “Conde de Valenciana,” Mexico City, Mexico; 2 Aris Vision Institute, Mexico City, Mexico

**Keywords:** thermography, cornea, laser surgery, excimer laser

## Abstract

**Purpose:**

To investigate the temperature of the cornea during treatment with the excimer laser using two platforms, Nidek EC-5000 and Schwind Amaris 750S.

**Methods:**

A prospective case series study was conducted in a reference center in Mexico City including patients aged 18 years or older who had any type of ametropia and underwent excimer laser refractive surgery. The patients had measurements of corneal temperature with an infrared camera before, during, and after ablation treatment. Results of prior corneal surface temperature, temperatures during excimer laser surgery, and delta temperature for each platform were analyzed and compared.

**Results:**

A total of 107 eyes were analyzed. Mean baseline temperature was 32.7 ± 1.03°C for the Nidek group and 31.5 ± 1.4°C for the Amaris group. Mean maximum temperature was 39.94 ± 1.3°C for the Nidek group and 35.6 ± 1.5 °C for the Amaris group. Delta temperature was higher in the Nidek group than in the Amaris group. There were statistically significant associations between treated micrometers, treated diopters, and time in the Nidek group and no such associations in the Amaris group.

**Conclusions:**

The different excimer laser devices used and the variety in the optical design, together with different software ablation algorithms, resulted in different levels of thermal loading; peak temperature rose in all measurements. Eyes treated with Nidek reached temperatures that doubled those found with Amaris.

**Translational Relevance:**

The correlation between Delta of temperature with defocus, depth, and treatment time is different regarding excimer laser generation.

## Introduction

Corneal temperatures in healthy individuals range from 31 to 37°C, being slightly colder in the center than in the periphery and colder than the rest of the tissues due to the cornea´s lack of vascularization and the constant evaporation of the tear film in its surface.^1^^,^^2^

Studies carried out in animal models show that thermal damage to the cornea is broadly related to the temperature and time of exposure to corneal tissue, including protein denaturation and destruction of keratocytes and endothelium, especially in those corneas exposed to temperatures above 45°C for 45 minutes, while at the same temperature of 45°C but exposed for 15 minutes, they demonstrated much greater tolerance and recovery.^3^

Excimer lasers used in corneal refractive surgery use a gaseous mixture of argon and fluorine. An inert gas, helium, serves to transfer energy. Under the effect of strong electric discharges (pulsed mode), the electrons of the argon–fluoride atoms move to a higher energy level; the excited atoms form an unstable molecule known as a “dimer.” The latter returns to its stable state, emitting high-energy photons with a wavelength of 193 nm. This type of corneal laser is suitable for various reasons, among which are weak penetration into adjacent tissues, minimal thermal effects, and a very smooth impact surface.^4^^–^^6^

The laser beam is modified by an optical system of prisms, masks, and mirrors to achieve its maximum homogenization, thus enabling it to be administered to the cornea, for which there are various strategies, depending on the manufacturer.^4^^,^^7^^,^^8^

The types of laser beams for refractive surgery are (1) the wide beam, being the oldest, and currently in disuse; (2) the slit scan, in which the beam is modified by a rotating diaphragm; and (3) the floating point, which uses only the center of the beam with a small area by means of high-frequency pulses that move according to the refractive requirement of the cornea to be treated; the third type theoretically generates the lowest ablation temperature.^9^^,^^10^

Infrared thermography provides a noninvasive temperature measurement of the ocular surface and an objective quantitative outcome.^11^ Purslow and Wolffsohn^12^ demonstrated that the temperature of the ocular surface measured by infrared thermography is mainly related to the tear film.

More contemporary is the use of thermography during refractive surgery. Betney et al.^13^ observed that ocular surface temperature (OST) increased during photorefractive keratectomy (PRK) to levels at which proteins can be denatured and that neither the depth of ablation nor the duration of the procedure correlated with OST. In contrast, Maldonado-Codina et al.^14^ found greater increases of OST for deeper treatments. Both studies suggest that the thermal load of the cornea may be involved in the haze that occurs in the postoperative period.

In view of the above findings, the aim of this study was to compare the thermal load and corresponding increase of corneal temperature during excimer laser ablation using two excimer laser platforms: Nidek EC-5000 (Nidek Instruments, Gamagori, Japan) and Schwind Amaris 750S (Schwind eye-tech-solutions, Kleinostheim, Germany). Thus, we focused on (1) evaluating the increase in corneal temperature during laser photoablation with different platforms (scanning slit and flying spot), (2) evaluating the increase in corneal temperature during laser photoablation with PRK and laser-assisted in situ keratomileusis (Lasik), (3) determining the temperature difference (Delta T), and (4) correlating the Delta T with treated micrometers, treated diopters, and time of ablation.

## Methods

A prospective, descriptive case series study was carried out in the Conde de Valenciana Institute of Ophthalmology and Aris Vision Institute of Ophthalmology in Mexico City. Inclusion criteria were that they were 18 years or older, having any type of ametropia, and undergoing refractive surgery with excimer laser. Patients who had excimer laser treatment for reasons different from ametropia were excluded. The study included 59 patients (107 eyes) who were assigned to two groups for the comparison of the excimer laser platforms investigated: the Nidek EC-5000 group and the Schwind Amaris 750S group. The corneal temperature of the patients was measured with an infrared camera before, during, and after ablation treatment. Prior corneal surface temperatures, temperatures during excimer laser surgery, and the delta temperature for each excimer laser platform were analyzed and compared.

The study protocol was approved by the Ethics Committees of the Conde de Valenciana Institute of Ophthalmology and Aris Vision Institute of Ophthalmology. Informed consent was obtained from the participants after explanation of the nature and possible consequences of the study. All study procedures were performed in accordance with the tenets of the Declaration of Helsinki.

The descriptive statistics used for this study were means and standard deviations (SDs). For the comparison of variables, the Kolmogorov–Smirnov test was used to assess the normal distribution of data; then variables were compared between the groups using the *t*-test. When the nonnormality criteria were met, the Wilcoxon rank test was used. The associations of variables were tested using the Pearson correlation coefficient. For statistical analysis, Stata Statistical Software for Windows version 15.1 (StataCorp, College Station, TX, USA) was used. *P* = 0.05 was considered statistically significant.

### Laser Systems and Software

Both lasers were calibrated before surgery by ablating a polymethylmethacrylate target for the Nidek platform and a polyethylene terephthalate (PET) foil for the Amaris platform. The parameters were adjusted to those commonly used in daily clinical practice (creep, 118 mJ /cm^2^; repetition frequency, 30 Hz; ablation rate, 0.6 μm/scan; pulse width, 20 ns). A hygrometer allowed measurement of the temperature and humidity of the surgery room in which the laser procedure for each eye was performed.

### Refractive Settings

The treatments were performed on different days by several cornea specialists for each group (Nidek and Amaris). Included were patients with hyperopic and myopic astigmatism. The different number of eyes measured for each group depended on the number of patients scheduled for each day. Only patients unbothered by their higher-order aberrations and with preoperative monocular corrected distance visual acuity of 20/20 or better were included. All treatments were uneventful, and no interruption of the pulse sequence occurred. In all treatments, the optical zone ranged from 5.5 to 7.0 mm.

### Setup for Thermodynamic Measurements

During the procedure, a FLIR ONE noncontact infrared detection camera (FLIR Systems, Tigard, OR, USA) was used to measure the temperature of the ocular surface. The camera employed a detection unit and a dynamic multispectral imaging system (MSX, Wilsonville, Oregón, Estados Unidos), which is the integrated processor that incorporates thermal video in real time with the definition of the visible spectrum. Under stabilized environmental conditions with a temperature of 23.3°C in the operating room, the infrared camera was connected directly to the mobile device with the corresponding application (FLIR ONE) and then mounted on a tripod—designed by the main researcher of this study—that fitted over the laser viewfinders and allowed the camera to be just above the eye. The camera was set at approximately 20 cm from the cornea (see experimental setup in Supplementary Fig. S1). The instrument detects energy in a wide dynamic range from −20°C to 120°C and displays images encoded by color palettes on the screen of the mobile device (iOS or Android) with a thermic resolution of 160 × 120 pixels, a thermal sensitivity of 70 mK, and a time constant of 12 ms at a frequency of 8.7 Hz. The analysis of the thermograms was done on a computer in the file formats .jpeg radiometer for images or MPEG-4 (MOV/MP4) for video. Each film clip of each treatment was divided into 1/8.7-second frames. The box or image showing the highest temperature during the treatment on the cornea was selected; this was done by first placing a box of 5 × 5 pixels on the corneal area. This box represented an area of approximately 1.7 mm^2^ in the cornea. Two zones were chosen: one on the forehead as a reference and the other one on the center of the cornea. During the entire measurement process and for every image, the maximum and minimum values, together with the mean and 95% confidence interval, were considered and evaluated over the area of interest. To evaluate the change in temperature, the temperature distributions immediately before ablation were analyzed. The extracted values were sequentially traced from one image to the next and individually for each patient. Spherocylindrical refractive values are presented with their defocus and spherical equivalent refraction. Mean values in degrees Celsius represent the change in the overall measured temperature points. Maximum temperature values demonstrate the maximal increase in degrees Celsius over all measurements and all measured points during the entire ablation process. In the Nidek group, in which spherocylindrical ablation took place in two moments, the cylinder was treated first, followed by spherical ablation, and the maximum temperature was selected between both ablations. The last measurement before ablation was determined to be the starting point for temperature values. All measurements made before and after the surgery related to this temperature showing either an increase or decrease. Following our institution's excimer laser refractive surgery protocol, we used, in all patients, cold balanced salt solution at 4°C after excimer laser ablation for stromal irrigation; 0.02% mitomycin C was used for 20 seconds in stroma in patients with PRK after excimer laser ablation. Temperature measurements were made before using these substances.

The operated patients were checked on their first day, first week, first month, and 3 months after surgery; a complete ophthalmologic examination was performed at each of these visits (visual acuity for each eye, refraction by autorefractometer for each eye, and graduation of haze for each eye using the corneal haze grading scale proposed by Fantes^21^ for patients undergoing PRK). The following postsurgical topical treatment scheme was used: 0.1% fluoromethalone acetate (Flumetol; Sophia Laboratories, Guadalajara, Jalisco, Mexico): one drop every 4 hours for 1 week, one drop every 8 hours for 1 week, and one drop every 12 hours for 1 additional week; moxifloxacin 0.5% (Vigamox; Alcon Laboratories, Fort Worth, TX, United States): one drop every 4 hours for 7 days; and lubricant sodium hyaluronate 0.4% (Lagricel PF; Sophia Laboratories): one drop every 3 hours for 3 months.

## Results

There were 59 patients involved in the study: 25 patients in the Schwind Amaris group (average age of 28.36 ± 5.6 years; 60% were women) and 34 in the Nidek group (average age of 28.88 ± 5.5 years; 59% were women); no statistical differences were found regarding age between groups (*P* = 0.724). A total of 107 eyes were analyzed: 66 eyes underwent surgery with the Nidek EC-5000 system and 41 eyes with the Schwind Amaris 750 system. In the Nidek group, 30 eyes had Lasik and 36 had PRK. In the Amaris group, 34 eyes had Lasik and 7 had PRK. The mean room temperature during surgeries was 22.3 ± 0.95°C, and the mean humidity was 39.1 ± 3.7%.

**Figure 1.**
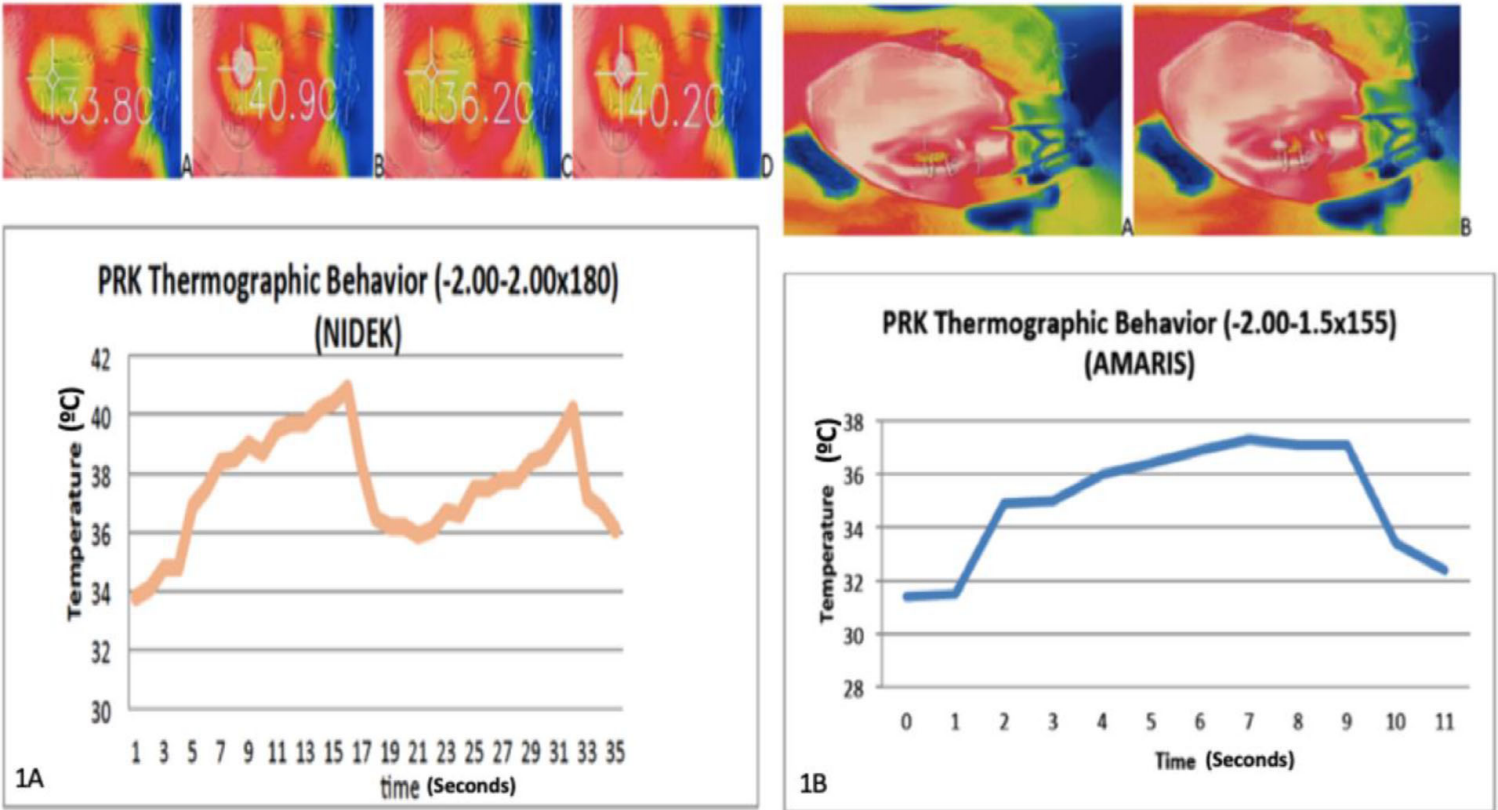
A, Thermographic behavior of the cornea in a patient who underwent PRK of −2.00–2.00 × 180 in the Nidek group. Temperature elevation of 7.1°C. Picture A is the basal temperature (33.8°C), picture B is the maximum temperature reached during cylinder treatment (40.9°C), picture C is the basal temperature before spherical treatment (36.2°C), and picture D is the maximum temperature reached during spherical treatment (40.2°C). The graphic below the pictures shows the two peaks of maximum temperature, the first one for cylinder treatment at 15 seconds and the second one for sphere treatment at 31 seconds. B, Thermographic behavior of the cornea in a patient who underwent PRK of −2.00–1.50 × 155 in the Amaris group. Temperature elevation of 5.5°C. Picture A is the basal temperature (31.8°C) and picture B is the maximum temperature reached during treatment (37.3°C). The graphic below the pictures shows the peak of maximum temperature reached at 7 seconds of treatment.

The thermographic behavior of both excimer laser platforms in patients with similar refractions is shown in Figures 1A and 1B for Nidek and Amaris, respectively.

The mean ± SD overall ocular surface temperature was 32.7 ± 1.03°C for the Nidek group and 31.5 ± 1.4°C for the Amaris group, after epithelial debridement or flap lift, with no statistical differences (*P* = 1.0). The parameters evaluated in each group are shown in Supplementary Table S1 and Table S2.

**Figure 2.**
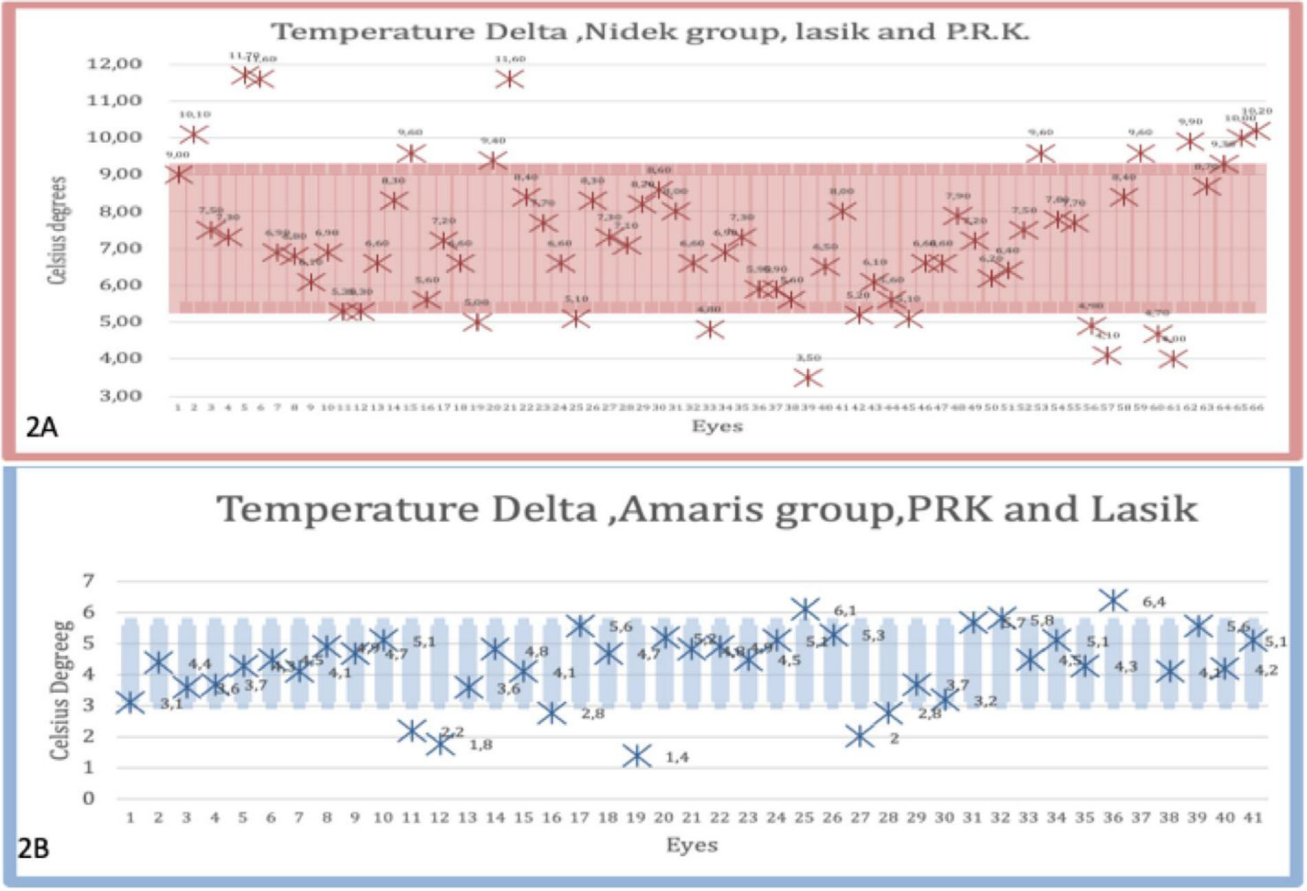
A, Delta T Nidek group, 66 eyes (mean, 7.27; variance, 3.45; SD, 1.87). B, Delta T Amaris group, 41 eyes (mean, 4.37; variance, 1.57; SD, 1.25).

The Delta (T) of the mean ± SD temperature for the Nidek group was 7.27 ± 1.87°C (maximum limit, 7.16; minimum limit, 5.39) compared to a mean ± SD of 4.36 ± 1.25°C (maximum limit, 5.62; minimum limit, 3.11) for the Amaris group, demonstrating a statistically significant difference (*P* = 0.0001) (Figs. 2A, 2B).

Within the Nidek group, 30 Lasik and 36 PRK-type surgeries were performed. The Delta (T) of the mean ± SD temperature for the Nidek-PRK group was 6.46 ± 1.20°C (maximum limit, 7.66; minimum limit, 5.25) compared to a mean ± SD of 7.34 ± 1.86°C (maximum limit, 9.20; minimum limit, 5.48) for the Nidek-Lasik group, demonstrating a statistically significant difference between these two groups (*P* = 0.0001).

Within the Amaris group, 34 Lasik and 7 PRK-type surgeries were performed. The Delta (T) of the mean ± SD temperature for the Amaris-PRK group was 4.08 ± 1.27°C (maximum limit, 5.36; minimum limit, 2.81) compared to a mean of 4.42 ± 1.26°C (maximum limit, 5.68; minimum limit, 3.16) for the Nidek-Lasik group, demonstrating a nonstatistically significant difference between these two groups (*P* = 0.6631).

All measurements showed an increase in temperature during laser ablation for both groups (see Supplementary Tables S3 and S4). The increase in temperature was higher in the Nidek group than in the Amaris group, with a mean ± SD maximum temperature of 39.9 ± 1.3°C and 35.6 ± 1.5°C, respectively (*P* = 0.032). Regarding the increase in corneal temperature during laser photoablation with PRK and Lasik, the Nidek group had higher loads and Delta T for both types of surgeries (*P* = 0.035). In relation to the length of time to complete the ablation, the Amaris and Nidek groups had an average time of 15 ± 6.5/21 ± 12 seconds, respectively.

In a relation 1:1 regarding defocus, both groups showed the same basal (mean) OST temperature before treatment, although maximum temperature and Delta T were higher in the Nidek group than in the Amaris group. These findings were statistically significant (*P* = 0.001) (Table).

**Table.** Temperature during Laser Corneal Refractive Surgery in 25 Eyes for Amaris and Nidek (1:1 Relationship) Pair by Same Defocus.

	Mean OST	Mean OST	Maximum T	Maximum T	Delta T	Delta T
Defocus	Amaris, °C	Nidek, °C	Amaris, °C	Nidek, °C	Amaris	Nidek
4.5	33.9	32.9	37	40	3.1	7.1
3.87	33	32.6	36.6	39.2	3.6	6.6
3.5	32.7	34.1	37	39.2	4.3	5.1
4.5	32.5	32.9	36.6	40	4.1	7.1
1.62	32.5	33.1	37	38	4.5	4.9
3.5	32.4	34.6	37.3	41.2	4.9	6.6
3.25	32.1	33	36.8	40.5	4.7	7.5
2.75	31.9	31.2	37	40.8	5.1	9.6
3	31.6	32.6	33.4	39.5	1.8	6.9
6.25	31.4	30.9	36.2	39.3	4.8	8.4
6.12	31.2	32.2	35.3	42.3	4.1	10.1
5.25	31.2	32.9	34	41.2	2.8	8.3
1.25	30.2	33.5	31.6	38.2	1.4	4.7
3.62	30	32.5	35.2	40	5.2	7.5
3.5	31	32.5	35.8	40.3	4.8	7.8
3.37	30.8	34.4	35.3	41	4.5	6.6
3.37	31	34.4	33.8	41	2.8	6.6
2.5	29.5	33.6	33.2	40.9	3.7	7.3
3.37	32.5	34.4	35.7	41	3.2	6.6
2.75	31.5	31.2	37.3	40.8	5.8	9.6
1.62	29.2	35	33.7	39.1	4.5	4.1
5.75	31.6	33.2	36.7	40.1	5.1	6.9
6.25	31.2	30.9	35.5	39.3	4.3	8.4
3.5	32.1	33.2	37.7	40.9	5.6	7.7
2.12	30.2	32.5	35.3	38.6	5.1	6.1

Mean = basal temperature.

**Figure 3.**
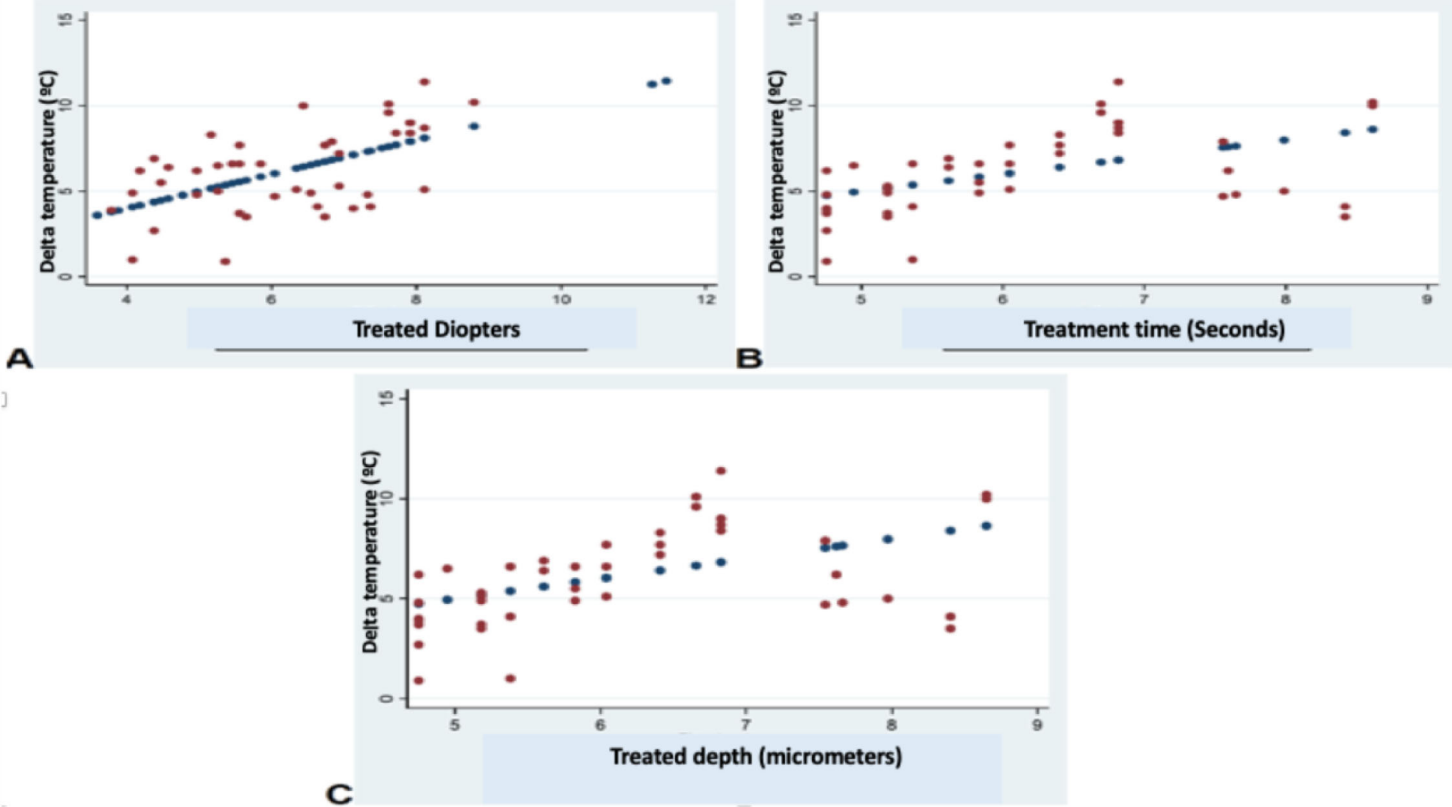
Associations between Delta temperature and treatment time, treated micrometers, and defocus using the Pearson correlation coefficient in cylinder treatment in the Nidek group. **A**, Delta T cylinder and treated diopters (y = 2.80 + 0.78; *R*^2^ = 0.55). **B**, Delta T cylinder and treatment time (y = 54.32 + 0.13; *R*^2^ = 0.47). **C**, Delta T cylinder and treated micrometers (y = 4.32 + 0.60; *R*^2^ = 0.47).

**Figure 4.**
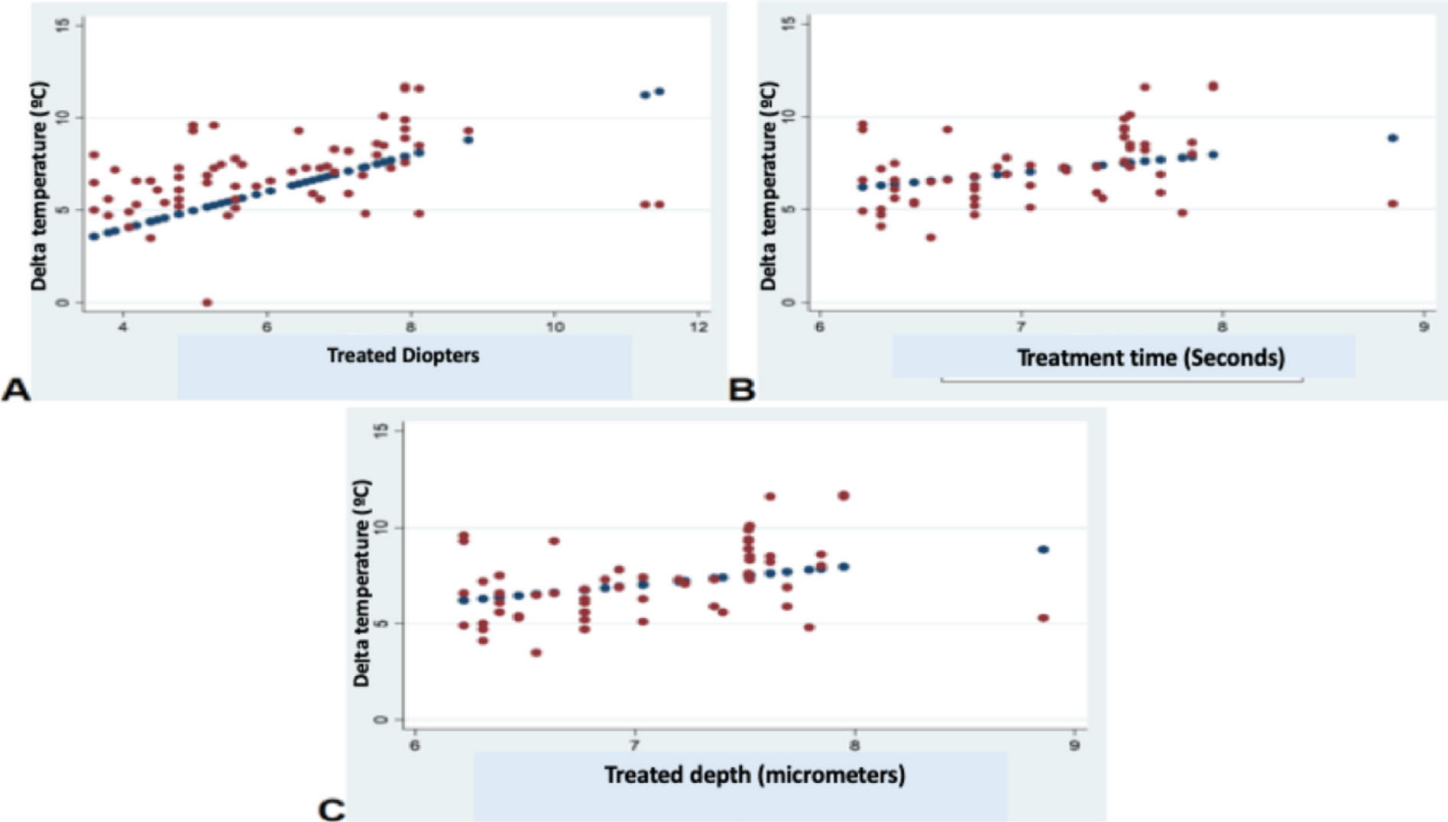
Associations between Delta temperature and treatment time, treated micrometers, and defocus using the Pearson correlation coefficient in sphere treatment in the Nidek group. **A**, Delta T sphere and treated diopters (y = 5.62 + 0.32; *R*^2^ = 0.35). **B**, Delta T sphere and treatment time (y = 5.97.32 + 0.053; *R*^2^ = 0.33). **C**, Delta T sphere and treated micrometers (y = 5.98 + 0.022; *R*^2^ = 0.33).

**Figure 5.**
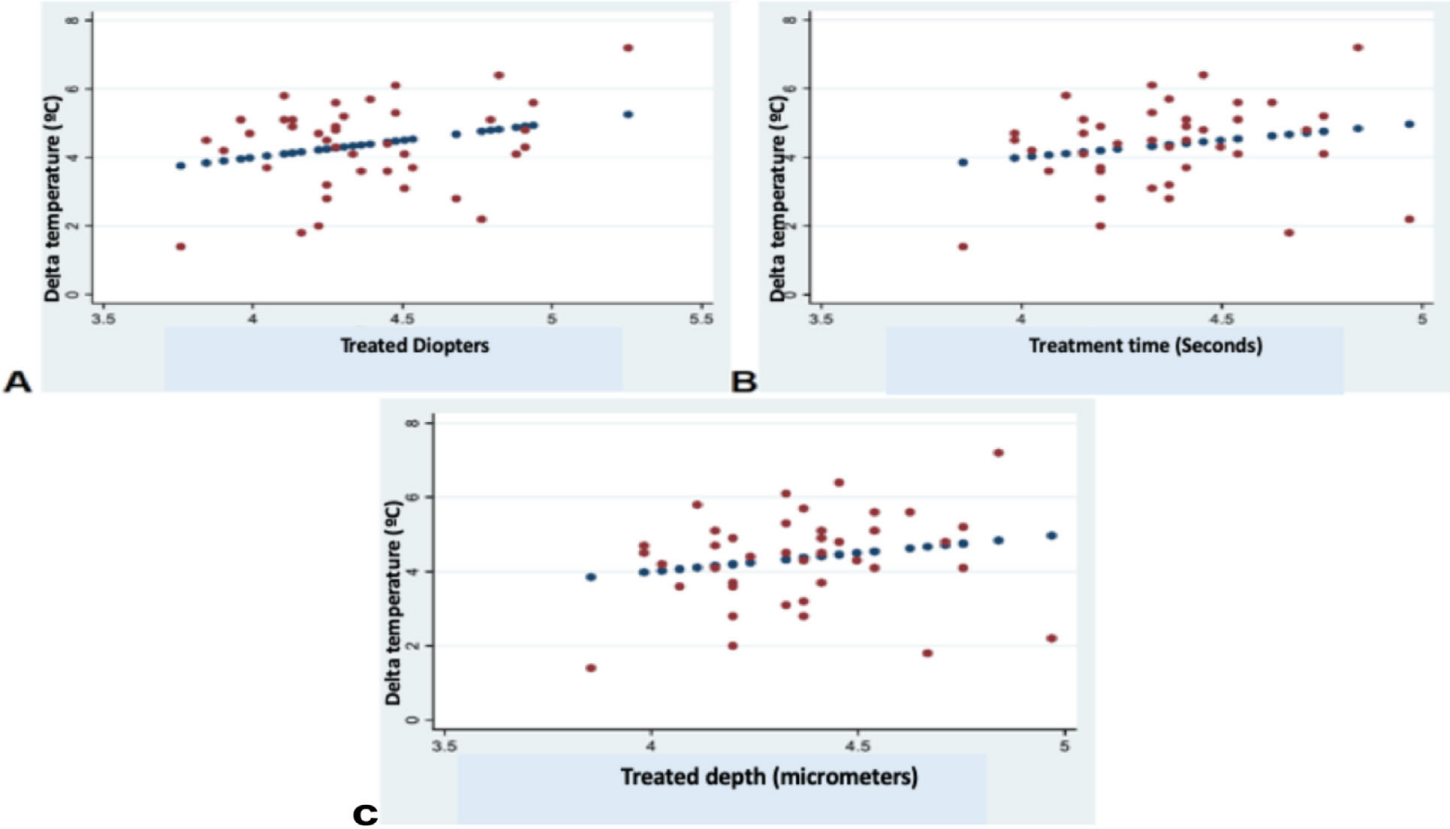
Associations between Delta temperature, treated diopters (defocus), time, and micrometers using the Pearson correlation coefficient in the Amaris group. **A**, Delta T and treated diopters (y = 3.47 + 0.23; *R*^2^ = 0.27). **B**, Delta T and time (y = 3.72 + 0.042; *R*^2^ = 0.19). **C**, Delta T and treated micrometers (y = 3.72 + 0.042; *R*^2^ = 0.19).

The assessment of the pairwise associations between Delta T and ablation depth (micrometers), treatment time, and defocus using the Pearson correlation coefficient showed, in the Nidek group, statistically significant associations between Delta T cylinder and ablation depth (micrometers), treatment time, and defocus (*P* = 0.001, *P* = 0.001, *P* = 0.01, respectively), as well as between Delta T sphere and ablation depth (micrometers), treatment time, and defocus (*P* = 0.001, *P* = 0.001, *P* = 0.01, respectively). In contrast, in the Amaris group, there were no statistically significant associations between Delta T and ablation depth (micrometers), treatment time, and defocus (*P* = 0.21, *P* = 0.21, *P* = 0.08, respectively) (Figs. 3–5).

For patients who underwent PRK, the degree of corneal haze was assessed on days 1, 7, 30, and 90. In the Amaris group, a mean ± SD degree of corneal haze of 0.57 ± 0.30 was recorded at day 1, 0.48 ± 0.23 at day 7, 0.32 ± 0.24 at day 30, and 0.13 ± 0.22 at day 90. In the Nidek group, a mean ± SD degree of corneal haze of 0.59 ± 0.27 was recorded at day 1, 0.50 ± 0.20 at day 7, 0.31 ± 0.24 at day 30, and 0.08 ± 0.19 at day 90. No statistically significant difference was found between the subgroups in any of the follow-up points (*P* = 0.75, *P* = 0.65, *P* = 0.79, *P* = 0.21).

## Discussion

The Nidek EC-5000 laser is a second-generation excimer laser that uses a slit microbeam for scanning and rotation that, after sending a laser shot through the slit, rotates 120° repeatedly until covering all 360°. The area of the slit is increased through an expanding slot, up to the maximum dimensions of 2 × 7 mm. In this system, the cylinder is treated first, and the sphere is treated seconds later.

The Schwind Amaris 750s laser employs a 750-Hz pulse frequency that reduces treatment time significantly. The automatic fluence level adjustment method promotes a balanced ratio between the total number of laser pulses and the energy delivered (80% of high fluence value treatment and 20% remaining of lower fluence). Its extremely small size (0.54 mm) and its super-Gaussian beam profile provide a smooth and tissue-saving ablation. The Amaris uses an eye tracker camera that monitors the position of the eye 1050 times per second.

The findings of corneal heating during excimer laser ablation are supported by this study. The different excimer laser devices used and the variety in the optical design, together with different software ablation algorithms, resulted in different levels of thermal loading. Peak temperature rose in all measurements, but the temperature for the Schwind Amaris 750S was lower than that measured for the Nidek EC-5000 and other published values.^13^^,^^14^ This is consistent with a preclinical study of porcine eyes, a clinical study of patients treated with the same Schwind Amaris model^15^^,^^16^ that we used, and a clinical study by Betney et al.,^13^ in which the Nidek EC-5000 was used for PRK. The setup of the mentioned studies was similar to ours, using a noncontact, infrared detector, but in those studies, the camera was mounted on a tripod with an angle of 45°, whereas in our study, the camera was above the eye. We want to emphasize that our findings and differences in corneal temperature during treatment do not suggest that one laser system is superior clinically to another; this was simply a descriptive study.

Why heat affects the fine structure of the cornea and, most important, at what temperature human corneal collagen begins to become altered is unclear. Bovine corneal collagen extracts have been demonstrated to undergo denaturation starting at 38.7°C and denaturing completely at 40°C.^17^

The importance of tissue temperature, as well as the time‒temperature relation, is well recognized in the process of tissue photothermal injury.^3^^,^^4^^,^^15^ This information may be useful during laser excimer laser surgery for its association with postoperative haze.

The literature reports that cooling the cornea before, during, or after PRK reduces postoperative haze.^18^^,^^19^ One prospective randomized study carried out on human eyes found that eyes undergoing cooling at PRK experienced less postoperative pain and less haze at 3 months compared with eyes with no cooling procedure done.^18^^,^^19^

Clearly, temperature plays some role in the postoperative wound-healing response, and its effect on PRK and Lasik deserves more thorough clinical and laboratory investigation. The use of a cooling substance during high cylinder treatments should be considered in patients to be treated with the Nidek system to avoid high temperatures.

Similar to previous publications, this study confirmed a corneal temperature increase. Maldonado-Codina et al.^14^ investigated the temperature changes occurring during PRK when performed at different ablation depths using noncontact, color-coded ocular thermography with an infrared detector during ablation in 19 bovine corneas. The authors observed an average temperature rise at the corneal surface of 8°C with a maximum rise of 9°C. Mrochen et al.^7^ investigated the influence of temporal and spatial spot sequences using a 1050-Hz high-repetition rate excimer laser. They found a relationship between ocular surface temperature increase and the amount of refractive correction. Using the most highly optimized scan, they observed a maximum temperature increase of 15°C in bovine corneas for the 9.00-diopter (D) myopia treatment and 11°C for 6.00-D hyperopia correction. During 50 μm of phototherapeutic keratectomy ablation, the thermal load heated the cornea by 11°C.

Algorithms for the newest generation of surgery equipment aim to minimize the thermal load in addition to optimizing correction of refraction in appropriate time with customized treatments, based on a sequential application of spatial distributed laser pulses.^8^^,^^20^ This is the reason that correlations regarding delta temperature and treatment time, depth, and treated diopters in the Amaris group are not significant.

Our limitations are that factors such as examination field, angle of examination, camera calibration, and heat radiations blockage are difficult to control and can influence results. In addition, the two peaks of treatments done with Nidek were different from the spherocylindrical ablation performed with Amaris.

In conclusion, this clinical study provides a more varied range of eyes that supports the evidence of elevated corneal temperature during refractive surgery with excimer laser and, to our knowledge, is the first study to compare two different generations of excimer laser platforms. Moreover, having the camera above the cornea ensures more accurate measurements because the camera is parallel to the cornea radiation emission surface, not angled at 45° as in previous studies. Finally, we suggest that temperature must be measured comparing wavefront and standard treatments with new-generation excimer laser platforms.

## Supplementary Material

Supplement 1

Supplement 2

Supplement 3

Supplement 4

Supplement 5
