# Supplementary material for: Thermographic Behavior of the Cornea During Treatment With Two Excimer Laser Platforms
Source: Transl Vis Sci Technol. 2021 Aug 24;10(9):27. doi: 10.1167/tvst.10.9.27 (PMC8399240; doi:10.1167/tvst.10.9.27)
Supplement: Supplement 1 [file tvst-10-9-27_s001.pdf]

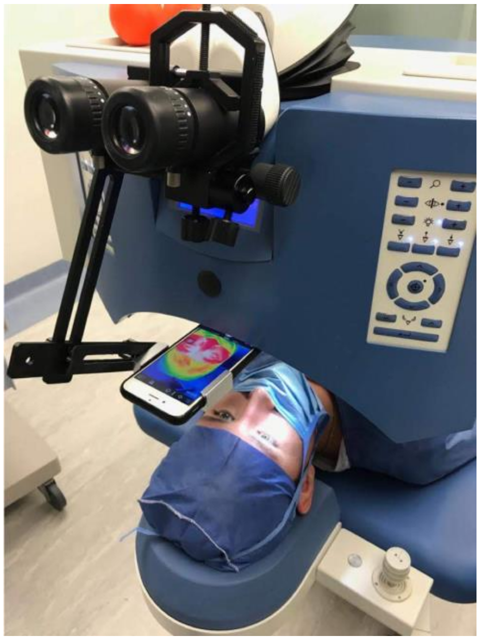

Figure S1. Experimental set-up. The infrared camera was connected directly to the mobile device with the corresponding application (FLIR ONE) and then mounted on a tripod—designed by the main researcher of this study—that fit over the laser viewfinders and allowed the camera to be just above the eye. The camera was at approximately 20 cm from the cornea.
