# Supplementary material for: Thermographic Behavior of the Cornea During Treatment With Two Excimer Laser Platforms
Source: Transl Vis Sci Technol. 2021 Aug 24;10(9):27. doi: 10.1167/tvst.10.9.27 (PMC8399240; doi:10.1167/tvst.10.9.27)
Supplement: Supplement 2 [file tvst-10-9-27_s002.pdf]

Table S1 Ablation Profile for Forty One Eyes That Underwent Laser Corneal Refractive Surgery with SHWIND AMARIS 750 System

Patient No/Eye	Refraction			Spherical Equivalent	Ablation Depth (micrometers)	Surgery
1/OS	-3.75	-1.5	179	-4.5	80.2	LASIK
2/OS	-3.75	-1	180	-4.25	72.93	LASIK
2/OD	-3.5	-0.75	180	-3.87	65.47	LASIK
3/OS	-3.5	-2.25	180	-4.62	86.93	LASIK
4/OS	-2.25	-2.5	160	-3.5	73.35	LASIK
5/OS	-0.5	-2.25	0	-4.5	43.12	LASIK
1/OD	-3.75	-1.5	3	-1.62	80.36	LASIK
6/OS	-3	-1	175	-3.5	61.82	LASIK
6/OD	-2.75	-1	175	-3.25	58	LASIK
5/OD	0.5	-4.5	180	-1.75	77.21	LASIK
7/OD	-3.25	-4.75	20	-5.62	123.96	LASIK
8/OD	-2.75	-0.5	4	-4.25	50.5	LASIK
9/OS	-2.25	-4	180	-3	97.03	LASIK
7/OS	-4.5	-3.5	165	-6.25	119.76	LASIK
10/OD	-5.5	-1.25	40	-6.12	103.85	LASIK
11/OS	-4.75	-1	7	-5.25	88.39	LASIK
12/OS	-5.5	-1.75	180	-6,37	110.31	LASIK
8/OS	-2	-0.5	172	-2.25	38.89	LASIK
13/OD	-1.25	0	0	-1.25	18.68	PRK
9/OD	-1.25	-4.75	180	-3.62	94.54	LASIK
14/OD	-2	-3	175	-3.5	74.58	PRK
14/OS	-1.25	-3.25	180	-2.87	67.16	PRK

15/OD	-2	-2.75	20	-3.37	74.07	LASIK
15/OS	-1	-3.75	175	-2.87	75.76	LASIK
16/OD	-3.5	1.75	15	4.37	79.98	LASIK
16/OS	-3.5	-1.75	165	-4.37	79.98	LASIK
17/OD	-2.75	-1	30	-3.25	52.8	LASIK
17/OS	-3	-0.75	165	-3.37	52.81	LASIK
18/OD	-1.75	-1.5	180	-2.51	47.88	PRK
19/OS	2.25	-2.25	160	-3.37	78.39	LASIK
20/OD	-3.25	-1.5	15	-4	72.66	LASIK
20/OS	-2	-1.5	155	-2.75	53.52	LASIK
21/OS	-1.25	-0.75	150	-1.62	29.84	PRK
22/OD	-5.5	-0.5	20	-5.75	92.91	LASIK
22/OS	-5.75	-1	0	-6.25	103.64	LASIK
23/OD	-5.25	1.25	155	-5.87	99.65	LASIK
23/OS	-6	-3.5	180	-7.75	141.05	LASIK
24/OD	-1.5	-4.5	15	-3.75	95.19	LASIK
24/OS	-1.5	-4	170	-3.5	87.2	LASIK
25/OD	-1.5	-0.75	20	-1.87	33.26	PRK
25/OS	-1.25	-1.75	145	-2.12	45.02	PRK
