# Supplementary material for: Thermographic Behavior of the Cornea During Treatment With Two Excimer Laser Platforms
Source: Transl Vis Sci Technol. 2021 Aug 24;10(9):27. doi: 10.1167/tvst.10.9.27 (PMC8399240; doi:10.1167/tvst.10.9.27)
Supplement: Supplement 3 [file tvst-10-9-27_s003.pdf]

Table S2 Ablation Profile for Sixty six Eyes That Underwent Laser Corneal Refractive Surgery with NIDEK EC-5000 System

Patient No/Eye	Refraction			Spheric equivalent	Ablation Depth(micrometers)	Surgery
1/OD	-5	-3	0	-6.5	112	LASIK
1/OS	-4.75	-2.75	0	-6.12	106	LASIK
2/OD	3	-0.5	170	2.75	49	LASIK
2/OS	2.5			2.5	35	LASIK
3/OD	-6.5			-6.5	91	LASIK
3/OS	-6.5			-6.5	91.5	LASIK
4/OD	-3			-3	42.25	PRK
4/OS	-2.5			-2.5	35.2	PRK
6/OD	-1.25	-1.75	175	-2.12	42.25	PRK
6/OS	-1.25	-1.5	175	-2	38.7	PRK
7/OD	-10	-2	10	-11	169	LASIK
7/OS	-10	-1.5	0	-10.75	161.9	LASIK
8/OD	-0.75	-2	180	-1.75	38.72	PRK
8/OS	-1.75	-2.5	170	3	65.48	PRK
9/OD	0,75	-4	170	1.25	66.89	LASIK
9/OS	-1.25			-1.25	17.6	PRK
10/OD	-1	-0.75	145	-1.37	24.64	PRK
10/OS	-2			-2	15.16	PRK
11/OD	-1			-1	14.08	PRK
11/OS	-5	-3	180	-6.5	112.66	LASIK
12/OD	-5.25	-3	170	-6.75	116.18	LASIK
12/OS	-5	-3	175	-6.5	112.66	LASIK
13/OD	-3.75	-2.5	5	5	88.02	LASIK
13/OS	-3.25	-1.25	175	-3.87	63.37	PRK
14/OD	-3.25	-0.5	10	-3.5	52.81	PRK
14/OS	-4.75	-1	10	-5.25	80.97	PRK
15/OD	-4.25	-1	145	-4.75	73.93	PRK
15/OS	-4	-1	165	-4.5	70.41	PRK
16/OD	-5.25	-0.5	0	-5.5	80.97	PRK
16/OS	-6			-6	84.5	PRK

17/OD	-6			-6	84.5	PRK
17/OS	2.25	-3.75	10	0.37	84.5	LASIK
18/OD	3.5	-4.75	170	1.12	116.18	LASIK
18/OS	-5.5	-0.5	10	-5.75	84.5	PRK
19/OD	-2.75	-0.75	170	-3.12	49.29	PRK
19/OS	-4.25	-1.25	30	-4.87	77.45	PRK
20/OD	-5.5			-5.5	77.45	PRK
20/OS	-2.5			-2.5	35.2	PRK
21/OD	-1.75	-0.5	10	-2	31.68	PRK
21/OS	0	-2	15	-1	28.16	LASIK
22/OD	0	-2	179	-1	28.16	LASIK
22/OS	-2.5			-2.5	35.2	PRK
23/OD	-2.5			-2.5	35.2	PRK
23/OS	-4.5	-1	170	-5	77.45	PRK
24/OD	-5.75	-2	5	-6.75	109.14	PRK
24/OS	-2.5	-1.75	180	-3.37	59.85	PRK
25/OD	-2.5	-2	5	-3.5	63.37	PRK
25/OS	-3.25	-3.75	170	-5.12	98.58	LASIK
26/OD	-4	-2.5	0	-5.25	91.54	LASIK
26/OS	-1.5	-0.5	135	-1.75	28.16	PRK
27/OD	-1.5	-1.5	10	-2.25	42.25	PRK
27/OS	1.25	-4.75	180	-1.12	84.5	LASIK
28/OD	1	-4.25	175	-1.12	73.93	LASIK
28/OS	-3	-1	145	-3.5	56.33	PRK
29/OD	-2.5	-2	40	-3.5	63.37	PRK
29/OS	-0.75	-1.75	175	-1.62	35.2	PRK
30/OD	-1	-1.25	0	-1.62	31.68	PRK
30/OS	-4.75	-3	15	-6.25	109.14	LASIK
31/OD	-4.75	-2.75	5	-6.12	105.62	LASIK
31/OS	-1	-0.5	20	-1.25	21.12	LASIK
32/OD	2	-2	160	1	56.33	LASIK
32/OS	-5	-3	0	-6.5	112.66	LASIK
33/OD	-5.25	-3	170	-6.75	116.18	LASIK
33/OS	0.75	-4	170	-1.25	66.89	LASIK
34/OD	-2	-5.25	10	-4.62	102.1	LASIK

34/OS	-5	-5.25	170	-7.62	144.35	LASIK
