# Supplementary material for: Thermographic Behavior of the Cornea During Treatment With Two Excimer Laser Platforms
Source: Transl Vis Sci Technol. 2021 Aug 24;10(9):27. doi: 10.1167/tvst.10.9.27 (PMC8399240; doi:10.1167/tvst.10.9.27)
Supplement: Supplement 4 [file tvst-10-9-27_s004.pdf]

Table S3 Temperature During Ablation for Sixty Six Eyes That Underwent Laser Corneal Refractive Surgery With NIDEK EC 5000.

Patient No./Eye	Defocus	Mean OST (ºC)	Maximum temperature (ºC)	Delta T (ºC)
1/OD	6.5	33	42	9
1/OS	6.12	32.2	42.3	10.1
2/OD	3.25	33	40.5	7.5
2/OS	2.5	33.6	40.9	7.3
3/OD	6.5	30.3	42	11.7
3/OS	6.5	30.2	41.8	11.6
4/OD	3	32.6	39.5	6.9
4/OS	2.5	32.2	39	6.8
6/OD	2.12	32.5	38.6	6.1
6/OS	2	33.4	40.3	6.9
7/OD	11	31.7	37	5.3
7/OS	10.75	31.7	37	5.3
8/OD	1.75	32.8	39.4	6.6
8/OS	3	34.5	42.8	8.3
9/OD	2.75	31.2	40.8	9.6
9/OS	1.25	32.6	38.2	5.6
10/OD	1.37	33.8	41	7.2
10/OS	2	32.5	39.1	6.6
11/OD	1	34	39	5
11/OS	6.5	31.2	40.6	9.4
12/OD	6.75	29.1	40.7	11.6
12/OS	6.5	31.4	39.8	8.4
13/OD	5	32.5	40.2	7.7
13/OS	3.87	32.6	39.2	6.6
14/OD	3.5	34.1	39.2	5.1
14/OS	5.25	32.9	41.2	8.3
15/OD	4.75	34.2	41.5	7.3
15/OS	4.5	32.9	40	7.1

16/OD	5.5	34	42.2	8.2
16/OS	6	31.4	40	8.6
17/OD	6	33.2	41.2	8
17/OS	4.12	31.8	38.4	6.6
18/OD	5.8	32.9	37.7	4.8
18/OS	5.75	33.2	40.1	6.9
19/OD	3.12	31.5	38.8	7.3
19/OS	4.87	33.1	39	5.9
20/OD	5.5	35.3	41.2	5.9
20/OS	2.5	33.5	39.1	5.6
21/OD	2	34.5	38	3.5
21/OS	1	32.6	39.1	6.5
22/OD	1	31.2	39.2	8
22/OS	2.5	32.8	38	5.2
23/OD	2.5	32.2	38.3	6.1
23/OS	5	34.5	40.1	5.6
24/OD	6.75	35	40.1	5.1
24/OS	3.37	34.4	41	6.6
25/OD	3.5	34.6	41.2	6.6
25/OS	5.12	32.9	40.8	7.9
26/OD	5.25	33	40.2	7.2
26/OS	1.75	33.9	40.1	6.2
27/OD	2.25	33.2	39.6	6.4
27/OS	3.62	32.5	40	7.5
28/OD	3.12	30.5	40.1	9.6
28/OS	3.5	32.5	40.3	7.8
29/OD	3.5	33.2	40.9	7.7
29/OS	1.62	33.1	38	4.9
30/OD	1.62	35	39.1	4.1
30/OS	6.25	30.9	39.3	8.4
31/OD	6.12	30.7	40.3	9.6
31/OS	1.25	33.5	38.2	4.7
32/OD	3	38.3	38.3	0
32/OS	6.5	30.2	40.1	9.9
33/OD	6.75	31.5	40.2	8.7

33/OS	2.75	32	41.3	9.3
34/OD	4.62	31.2	41.2	10
34/OS	7.62	31.6	41.8	10.2
