# Supplementary material for: Thermographic Behavior of the Cornea During Treatment With Two Excimer Laser Platforms
Source: Transl Vis Sci Technol. 2021 Aug 24;10(9):27. doi: 10.1167/tvst.10.9.27 (PMC8399240; doi:10.1167/tvst.10.9.27)
Supplement: Supplement 5 [file tvst-10-9-27_s005.pdf]

Table S4 Temperature During Ablation of forty One Eyes That Underwent Laser Corneal Refractive Surgery With SCHWIND AMARIS 750 system.

Patient No./Eye	Defocus	Mean OST (ºC)	Maximum temperature (ºC)	Delta T (ºC)
1/OS	4.5	33.9	37	3.1
2/OI	4.25	33.3	37.7	4.4
2/OD	3.87	33	36.6	3.6
3/OI	4.62	32.8	36.5	3.7
4/OI	3.5	32.7	37	4.3
5/OI	4.5	32.5	37	4.5
1/OD	1.62	32.5	36.6	4.1
6/OI	3.5	32.4	37.3	4.9
6/OD	3.25	32.1	36.8	4.7
5/OD	2.75	31.9	37	5.1
7/OD	5.62	31.6	33.8	2.2
8/OD	4.25	31.6	33.4	1.8
9/OI	3	31.6	35.2	3.6
7/OI	6.25	31.4	36.2	4.8
10/OD	6.12	31.2	35.3	4.1
11/OI	5.25	31.2	34	2.8
12/OI	6.37	30.8	36.4	5.6
8/OI	2.25	30.4	35.1	4.7
13/OD	1.25	30.2	31.6	1.4
9/OD	3.62	30	35.2	5.2
14/OD	3.5	31	35.8	4.8
14/OI	2.87	32.2	37.1	4.9
15/OD	3.37	30.8	35.3	4.5
15/OS	2.87	30.2	35.3	5.1
16/OD	4.37	31.4	37.5	6.1

16/OS	4.37	32	37.3	5.3
17/OD	3.25	31.5	33.5	2
17/OS	3.37	31	33.8	2.8
18/OD	2.51	29.5	33.2	3.7
19/OS	3.37	32.5	35.7	3.2
20/OD	4	30.3	36	5.7
20/OS	2.75	31.5	37.3	5.8
21/OS	1.62	29.2	33.7	4.5
22/OD	5.75	31.6	36.7	5.1
22/OS	6.25	31.2	35.5	4.3
23/OD	5.87	32.3	38.7	6.4
23/OS	7.75	30.7	37.9	7.2
24/OD	3.75	32.2	36.3	4.1
24/OS	3.5	32.1	37.7	5.6
25/OD	1.87	31	35.2	4.2
25/OS	2.12	30.2	35.3	5.1
